# Optimizing bacterial enrichment procedures to improve mosquito midgut microbiome research

**DOI:** 10.1186/s42523-026-00614-0

**Published:** 2026-08-17

**Authors:** Hans Schrieke, Blandine Trouche, David Kerrigan, Emilie Pondeville, Julie Reveillaud

**Affiliations:** 1 Mivegec, University of Montpellier, INRAE, CNRS, IRD, Montpellier, France; 2 MRC-University of Glasgow Centre for Virus Research, Glasgow, G61 1QH Scotland, UK

**Keywords:** Midgut microbiota, Host contamination, Bacterial enrichment and profiling

## Abstract

Mosquitoes are major vectors of infectious diseases, with their midgut microbiota playing a crucial role in host development, reproduction, and pathogen interactions. However, characterizing midgut microbial communities remains challenging because host DNA often dominates metagenomic libraries, reducing the resolution of bacterial community profiling and hindering the recovery of microbial genomes. To evaluate strategies for bacterial DNA enrichment in this context, we compared three DNA extraction protocols applied to pooled *Aedes aegypti* midguts: (i) the QIAGEN DNeasy Blood and Tissue kit without filtration (DNeasy NF), (ii) the same kit preceded by a 5 μm filtration step to reduce eukaryotic material (DNeasy F), and (iii) the QIAGEN QIAamp Microbiome kit, which combines differential host cell lysis and enzymatic DNA depletion. Each method was assessed using qPCR-based quantification of bacterial and host DNA, SSU rRNA-based taxonomic profiling of metagenomic reads, and Metagenome-Assembled Genomes (MAGs) reconstruction using advanced binning approaches. Our results show that while the Microbiome kit provided the highest bacterial enrichment and captured a large fraction (> 85%) of the bacterial taxa detected with the other kits, it yielded a higher relative abundance of *Gammaproteobacteria*. This occurred without a loss of overall diversity, suggesting improved recovery of microbial taxa rather than a compositional bias. The filtration step associated with the DNeasy kit significantly reduced host DNA but also lowered bacterial material, allowing MAG reconstruction, though less efficiently than the Microbiome kit. Despite this, the DNeasy methods allowed recovering a broader range of bacterial phyla with a more even distribution across taxa, while each extraction method also detected a unique set of NTUs. Overall, a combination of DNA extraction methods may be necessary to optimize microbiome analysis in mosquito midguts, facilitating a more comprehensive understanding of microbial diversity and its potential applications in vector control strategies.

**Supplementary Information:**

The online version contains supplementary material available at https://doi.org/10.1186/s42523-026-00614-0.

## Background

The mosquito midgut harbors a diverse range of microbes that play a crucial role in host physiology and development [1, 2]. Gut bacteria influence mosquito fitness and development [3–7], and several taxa (including *Wolbachia*, *Serratia*, and *Asaia*) have been shown to modulate vector competence and pathogen transmission [8–24]. Exploring this community, and particularly the fraction of microbes that are difficult to cultivate, is necessary for a better understanding of mosquito biology and to improve biocontrol strategies. While culture-based and amplicon-based approaches have provided much of our current knowledge of mosquito-associated bacteria [1, 25], they have known limitations: culture recovers only a fraction of the community, and amplicon sequencing can under-detect taxa through primer biases [26] and offers limited genomic resolution [27]. Shotgun metagenomics circumvents several of these limitations [28] and is now commonly used to explore the diversity and function of microbial communities. However, it introduces its own challenge as host-associated DNA can overwhelmingly dominate sequencing libraries, biasing bacterial detection and impairing genome reconstruction [29]. This challenge is especially acute in mosquito midgut samples, where bacterial biomass is inherently low relative to host tissue. Furthermore, metagenomic approaches are highly sensitive to contamination from foreign DNA that can be introduced by various sources including laboratory reagents, extraction or PCR kits also referred to as the ‘kitome’ [30–35]. Attempting to enrich bacterial material while controlling for these sources of bias is therefore a prerequisite for reliable midgut microbiome analyses.

In this study, we aimed to optimize and investigate the efficiency of DNA extraction methods in enriching bacterial material and enhancing microbiota analysis in pooled midguts from *Aedes aegypti* mosquitoes. We compared bacterial DNA enrichment using the QIAGEN DNeasy Blood and Tissue kit (with and without a filtration step to reduce eukaryotic material) and the QIAGEN QIAamp Microbiome kit, originally intended to enrich bacterial DNA by host depletion (without filtration). We quantified the relative abundance of bacterial and host DNA using qPCR targeting the 16S rRNA gene and the *RpS30* mosquito gene, respectively, for each condition. Then, we reconstructed MAGs from each sample and compared the abundance and diversity of bacterial genomes obtained under each condition. We present the results of these investigations and discuss how DNA extraction methods may complement each other, suggesting that an integrated approach may be necessary to optimize the analysis of microbiota in midgut tissues.

## Methods

### Sample collection and extraction

We collected adult females from the *Aedes aegypti* Paea strain (a generous gift from Dr Failloux, Institut Pasteur, France), a laboratory colony originating from French Polynesia and maintained for 450 to 500 generations. Adult *Aedes aegypti* from insectary laboratory colonies have been shown to harbor highly similar midgut bacterial microbiomes [36], making this long-established laboratory colony well-suited for a methodological comparison. Specimens were reared at 28 °C and 80% humidity conditions under a 12 h light/dark cycle as described in [37]. We followed a similar method as in [38] to prepare samples. Briefly, we anesthetized *Aedes aegypti* adult females by exposing them to 4 °C for 2 min. We placed them for three minutes in 1 mL of 70% ethanol to remove surface contaminants followed by three one-minute washes in 1 mL of sterile 1X Phosphate-Buffered-Saline solution (PBS). Subsequently, we transferred specimens one by one into a sterile dissection cup and dissected their midguts using sterilized forceps into sterile PBS 1X. Dissected organs were then rinsed in a 200 µl solution of sterile PBS 1X and immediately processed. For each extraction method, we prepared three samples of five midguts (R1, R2, R3 replicates for DNeasy NF, DNeasy F and Microbiome kit samples) and three blank samples as described below. Pools of five midguts were chosen to ensure sufficient DNA yield across all three extraction protocols, as the filtration step (DNeasy F) and the Microbiome kit both incur additional sample losses relative to direct extraction. Pooling also reduces stochastic biases associated with low-input, host-dominated samples [33, 39, 40]. Single-organ analyses were a deliberate alternative we chose not to pursue, as individual mosquito midguts can yield insufficient bacterial DNA for robust comparison across methods [41]. We note that individual-level variation is therefore not accessible in this dataset, and that conclusions are drawn at the pooled-sample level. Larger pools could have provided more biomass and possibly improved detection of low-abundance taxa, yet at the cost of fewer independent replicates per method; larger pool sizes may represent a relevant direction for follow-up work.

We tested three DNA extraction methods to compare bacterial enrichment defined here as the bacterial-to-host DNA ratio achieved by each method: (i) DNeasy NF: Midguts (or 20 µl of sterile PBS 1X for the blanks) were transferred into tubes containing sterile glass microbeads (0.5 mm; Sigma-Aldrich) and 180 µl of QIAGEN ATL buffer supplemented with 20 mg/mL lysozyme (to lyse the cell walls of gram-positive bacteria). Samples were homogenized using a Precellys 24 (Bertin Technologies) at 6000 rpm for 30 s and DNA from supernatant was extracted using the QIAGEN DNeasy Blood and Tissue kit. (ii) DNeasy F: Midguts were placed in a cup in 1.5 mL PBS 1X and opened longitudinally with forceps. The midguts with PBS (or PBS alone for the blanks) were transferred into a tube, shaken for 15 min at 300 rpm, and filtered through a 5 μm filter (Whatman Puradisc) to achieve host DNA depletion by physical separation, retaining mosquito midgut tissue on membranes. The 5 μm pore size allows passage of bacterial cells (typically 0.5 to 2 μm) while retaining mosquito tissue debris and eukaryotic cell nuclei. This pore size was chosen following protocols adapted on related arthropod microbiota [42]. To note, alternative pore sizes were not systematically tested in this study and may warrant further optimization. The filtrate was then centrifuged at 5000 rpm for 15 min, supernatant removed to leave 20 µl to which 180 µl of QIAGEN ATL buffer supplemented with 20 mg/mL lysozyme and sterile glass microbeads (0.5 mm; Sigma-Aldrich) were added. Samples were homogenized using a Precellys 24 (Bertin Technologies) at 6000 rpm for 30 s before DNA extraction using the QIAGEN DNeasy Blood and Tissue kit. And (iii) QIAGEN QIAamp Microbiome kit: Midguts were placed in a cup in 0.5 mL PBS 1X and cut into small pieces with sterile micro scissors. The midguts with PBS (or PBS alone for the blanks) were transferred into a tube, and DNA was extracted using the QIAGEN QIAamp Microbiome kit. All samples were eluted in 35 µl of nuclease-free water, and their concentration measured with a NanoDrop (ThermoScientific). Samples were aliquoted by 10 µl and stored at 4 °C until qPCR or -20 °C until sequencing.

### Real-time quantitative PCR (qPCR)

To quantify the relative abundance of bacterial and host DNA in samples from each DNA extraction procedure, qPCR assays were run with the comparative CT (cycle threshold) method using the QS3 Real Time PCR System (Applied Biosystems) and the Fast SYBR Green Master Mix method (Thermo Fisher Scientific) according to the manufacturer’s protocol. Bacterial DNA was quantified using primers amplifying the 16S rRNA gene from Wei et al. 2017 [43] (16SF 5’-TCCTACGGGAGGCAGCAGT-3’, 16SR 5’-GGACTACCAGGGTATCTAATCCTGTT-3’) and the mosquito host DNA was quantified using *RpS30* primers amplifying the *RpS30* ribosomal protein mosquito gene (*RpS30* qFOR 5’-CACTGCTGGGAGGTAAGGTG-3’, *RpS30* qREV 5’-GCGAAGTTGCGGTTGTACTG-3’, designed in this study). CT values were tested for normality (Shapiro-Wilk test) and analyzed by one-way (*RpS30*) or two-way (16S) ANOVA followed by a Tukey’s multiple comparison test. To analyze the bacterial enrichment between the different DNA extraction methods, 16S levels were normalized using *RpS30* according to the Taylor method [44] to obtain a geomean of RQ = 1 for the control group (DNeasy NF) and relative RQ values for every other group. Log2 values were tested for normality (Shapiro-Wilk test) and used to perform a one-way ANOVA followed by a Tukey’s multiple comparison test. Geomeans were used to calculate fold changes. Individual CT values are reported in Table S1 and statistical analyses were performed using GraphPad Prism v9.3.1 (GraphPad Software, San Diego, CA, USA).

### Sequencing

We performed all sequencing steps at the MGX sequencing platform in Montpellier (France). Briefly, DNA-seq libraries were prepared using the Illumina DNA Prep Kit (Illumina, San Diego, CA, USA). The genomic DNA was tagmented using the Bead-Linked Transposomes (BLT), which tag DNA fragments with adapter sequences. Tagmented DNA was then amplified using a limited-cycle program. The PCR step added Index 1 (i7), Index 2 (i5) and sequences required for sequencing cluster generation. The size distribution of the resulting libraries was monitored using a Fragment Analyzer with the High Sensitivity NGS kit (Agilent Technologies, Santa Clara, CA, USA) and the libraries were quantified using the KAPA Library quantification kit (Roche, Basel, Switzerland). Libraries were denatured using NaOH, neutralized with Tris-HCl, and diluted to 150 pM. Clustering and sequencing were performed on a NovaSeq 6000 (Illumina, San Diego, CA, USA) using the paired-end 2 × 150 nt protocol on 1 lane of an S4 flow cell. Image processing and base calling were performed using the NovaSeq Control Software and Real-Time Analysis component. Demultiplexing was performed using Illumina’s conversion software (bcl2fastq 2.20.0.422). Finally, a preliminary data quality assessment was done using FastQC (v0.11.9) from the Babraham Institute and the Illumina software SAV (Sequence Analysis Viewer). Potential contaminants were investigated with the FastQ Screen software from the Babraham Institute.

### Taxonomic profiling and estimation of host contamination based on SSU rRNA sequences

To estimate eukaryotic contamination (defined here as the residual fraction of eukaryotic, mostly host-derived, reads among reads mapped to SSU rRNA sequences) in our samples, we used phyloFlash v.3.4.2 [45] with “--readlength 150” and “--almosteverything” flags. This approach extracts small-subunit rRNA (SSU rRNA) from short reads by mapping them to the SILVA SSU Ref database version 138.1 [46] and reconstructs full-length SSU rRNA using SPAdes [47]. The analysis provides a comprehensive taxonomic summary integrating short reads, reconstructed full-length SSU rRNA and unassembled sequences to identify Nearest Taxonomic Units (NTUs). Of note, unlike Operational Taxonomic Units (OTUs) and Amplicon Sequence Variants (ASVs) which are derived from gene-marker-based approaches (e.g., 16S rRNA amplicon sequencing) and provide higher taxonomic resolution, NTUs in phyloFlash are assigned by directly mapping reads to a reference database. This method relies on database completeness and accuracy rather than *de novo* sequence clustering, making NTUs less precise but useful for rapid taxonomic profiling in metagenomic datasets. We used a custom R [48] script to extract and plot the percent of reads assigned to bacterial and eukaryotic SSU rRNA sequences. Moreover, we used R and the phyloFlash taxonomic assignment results to generate a composition plot displaying the percent of read distribution by Phylum in our samples for each DNA extraction method. To further investigate host contamination, we extracted eukaryotic reads identified by phyloFlash and mapped them onto the *Aedes aegypti* reference genome (genome assembly AaegL5.0, NCBI ID: GCF_002204515.2) using Bowtie2 v2.5.4 [49]. This enabled us to specifically estimate the proportion of host-derived sequences in our samples and complement the taxonomic information provided by phyloFlash. We assessed the influence of DNA extraction methods on (i) eukaryotic and host contamination and (ii) bacterial diversity after quantifying the number of NTUs per sample at the Order level, using a one-way ANOVA followed by a Tukey post-hoc multiple comparison. In addition, we estimated the effect of extraction method on overall bacterial community composition using Non-Metric Multidimensional Scaling (NMDS) based on Bray-Curtis dissimilarity distance and tested statistical differences using PERMANOVA (adonis2 function of the vegan R package v2.7-3 [50], 999 permutations). We verified homogeneity of variances across groups by conducting a permutational test for homogeneity of multivariate dispersions (betadisper function of the same package).

### Differential abundance analysis

To statistically compare bacterial composition between extraction methods, phyloFlash read counts (Table S2) were analyzed at the same taxonomic level as the composition profile (Phylum, with *Proteobacteria* resolved to class). Reads not resolved at that level were grouped as “Unassigned bacteria”, and taxa were retained only if detected in at least three samples with ≥ 50 reads overall. Differences between extraction methods were assessed with two complementary approaches: DESeq2 [51] v1.46.0 (negative binomial model) and ALDEx2 [52] v1.38.0 (compositional method with a centered log-ratio transformation), each with Benjamini-Hochberg correction. Given the pooled design and three replicates per method, these analyses are exploratory.

### Metagenomics workflow

We used anvi’o v7.1 [53, 54] to perform metagenomic analyses and more specifically the metagenomic snakemake [55] workflow from the quality filtering short reads to the complete merged anvi’o profile databases for each sample separately as in [41]. All the parameters used to set the snakemake workflow are reported in a “contig.json” available in Zenodo (see Data Availability section). Briefly, for each sample, we quality-filtered short reads using the “iu-filter-quality-minoche” anvi’o program with default parameters and trimmed the quality filtered short reads using the BBDuk program from the BBMap [56] v.38.93 tool (adapter sequence CTGTCTCTTATACACATCT and ktrim = r, k = 19, mink = 9, hdist = 1, tpe, tbo, t = 20 parameters). Additionally, we removed *Aedes aegypti* reads using the following steps: (1) masking low-complexity sequences (entropy < 0.7) of the *Aedes aegypti* reference genome (genome assembly AaegL5.0) with BBMask [56], and (2) removing sequences from the sample that matched this masked reference genome using a combination of Bowtie2 v2.5.4 [49] and the iu-remove-ids-from-fastq function of the anvi’o program. Then, we assembled them into contigs (with a length > 1000 nt) using metaSPAdes [57] v3.14.1 and performed read recruitment by mapping short reads from each sample on corresponding contigs using Bowtie2. We generated the anvi’o contig databases for each sample using the “anvi-gen-contigs-database” program which computed k-mer frequencies and identified open reading frames (ORFs) using Prodigal [58] v2.6.3. To identify Single-copy Core Genes (SCGs), we searched the hidden Markov models (HMMs) from various sources (Bacteria_71 [59], Archaea_76 [59], Protista_83 [60] collections) using the “anvi-run-hmms” program. For functional annotation, we searched contigs against COG20 [61] and KEGG [62] databases using the “anvi-run-ncbi-cogs” and “anvi-run-kegg-kofams” programs, respectively. Then, we computed the coverage at each nucleotide position and statistics for each metagenome assembly based on the BAM files using the “anvi-profile” program and we merged them into final anvi’o profile databases using the “anvi-merge” program. After the metagenomics workflow, we used CONCOCT [63] v1.1.0 to group contigs into bins for each sample and CheckM [64] v1.1.3 to estimate their quality (completeness, redundancy). Finally, we performed taxonomic assignment of the Metagenome-Assembled Genomes (MAGs) obtained using the GTDB-Tk [65] v2.4.0 classifier (based on GTDB release 220 [66]). To note, we intentionally avoided co-assembling reads from different extraction methods to preserve the integrity of the methodological comparison and to prevent genomes from being reconstructed using reads originating from multiple extraction conditions. We favoured this conservative strategy while acknowledging that combining replicates could increase MAG recovery, particularly for DNeasy F.

## Results

### Bacterial enrichment estimates using qPCR

**Fig. 1:**
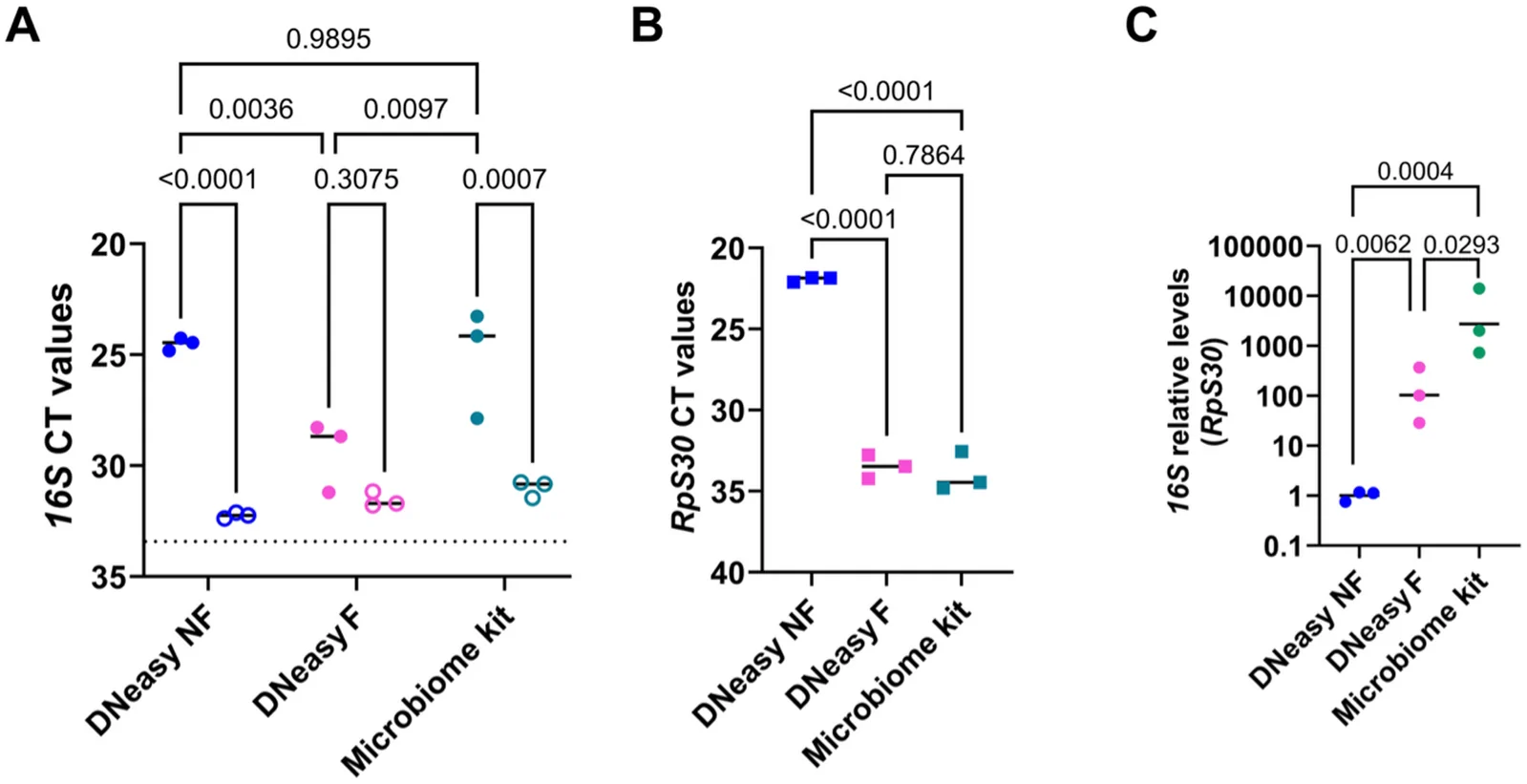
Bacterial enrichment between different DNA extraction methods. 16S rRNA gene (bacteria) and *RpS30* gene (mosquito) relative levels were quantified by qPCR for each DNA extraction method: DNA extracted using QIAGEN Blood and Tissue kit without filtration (DNeasy NF), filtration and QIAGEN Blood and Tissue kit (DNeasy F), no filtration and QIAGEN Microbiome (Microbiome kit). *N* = 3 samples of 5 midguts or 3 blank samples per group. Exact *p* values are shown. (**A**) 16S CT values. Plain circles: midgut samples; empty circles: blank samples. The black bars display the medians. The dotted line represents the PCR negative template control. Data were analyzed with two-way ANOVA followed by a Tukey’s multiple comparison test. (**B**) *RpS30* CT values. The black bars display the medians. Data were analyzed with one-way ANOVA followed by a Tukey’s multiple comparison test. (**C**) 16S rRNA gene relative levels normalized to the *RpS30* gene and to the control group (DNeasy NF, RQ geomean set to 1). The black bars display the geomeans (used to calculate fold changes). Log2 values were used to perform a one-way ANOVA followed by a Tukey’s multiple comparison test

To explore methods for enriching bacterial material and improving microbiota analysis in midgut tissue from *Aedes aegypti* mosquitoes, we extracted DNA from pools of midgut samples using three different methods: (i) using the QIAGEN DNeasy Blood and Tissue kit on whole midgut samples (control; DNeasy NF samples); (ii) using the QIAGEN DNeasy Blood and Tissue kit on filtrates (< 5 μm size) from opened midgut samples to achieve host DNA depletion by physical separation, retaining mosquito tissue debris and eukaryotic cell nuclei on the membrane while allowing bacterial cells to pass through (DNeasy F samples), and (iii) the QIAGEN QIAamp Microbiome kit, originally intended to enrich bacterial DNA by differential lysis of host cells and subsequent enzymatic digestion of host DNA (Microbiome kit samples). We then quantified by qPCR the relative abundance of bacterial and mosquito host material using the 16S rRNA gene and the *RpS30* mosquito gene, respectively, in each midgut sample and their respective blank controls. We observed that the bacterial abundance (16S, Fig. 1A, Table S1) was similar between DNeasy NF and Microbiome kit midgut samples (Tukey post-hoc, p value = 0.9895, Fig. 1A), while it was significantly lower in DNeasy F midgut samples (mean CT difference of ~ 4–5 cycles; Tukey post-hoc, p value = 0.0036 vs. DNeasy NF; p value = 0.0097 vs. Microbiome kit, Fig. 1A). The 16S CT values measured in blank samples were close to the PCR no-template control (Fig. 1A, dotted line), indicating a low background bacterial signal in the blanks. Blank CT values were significantly higher than those of the corresponding midgut samples for DNeasy NF (p value < 0.0001) and Microbiome kit (p value = 0.0007), consistent with a genuine bacterial signal in the midgut samples. For DNeasy F, blank and midgut CT values did not differ significantly (p value = 0.3075), consistent with the lower bacterial signal recovered with this method. In terms of mosquito host material (*RpS30*, Fig. 1B), we found that both DNeasy F and Microbiome kit samples had significantly lower mosquito DNA levels compared to DNeasy NF samples (both p values < 0.0001). We then normalized the 16S relative levels to the *RpS30* relative levels to determine the relative bacterial enrichment between extraction methods (Fig. 1C). The Microbiome kit samples showed a higher bacterial enrichment than DNeasy F samples (~ 27-fold enrichment; Tukey post-hoc, p value = 0.0293, Fig. 1C) and DNeasy NF samples (~ 2700-fold; Tukey post-hoc, p value = 0.0004; Fig. 1C), reflecting both the higher bacterial signal relative to DNeasy F (Fig. 1A) and the marked host DNA depletion relative to DNeasy NF (Fig. 1B). DNeasy F samples also showed a significant enrichment relative to DNeasy NF (~ 100-fold; Tukey post-hoc, p value = 0.0062; Fig. 1C).

### Bacterial enrichment and taxonomic profiling estimates using short subunit (SSU) rRNA extracted reads

We sequenced an average total of 238,879,191 raw paired-end reads across all samples (ranging from 203,604,476 to 276,466,122) (Table S3). Quality filtering and trimming of raw reads resulted in an average of 226,828,309 paired-end reads per sample (Table S3).

To estimate eukaryotic contamination, we used phyloFlash to map filtered reads onto SSU rRNA sequences and annotate them using the SILVA 138.1 database. Here, the SSU mapping ratio refers to the fraction of total reads that map to SSU rRNA sequences. On average, DNeasy NF resulted in 285,712 mapped SSU read pairs per sample with a mean mapping ratio of 0.12%, whereas DNeasy F yielded 196,826 mapped SSU read pairs per sample with a lower mapping ratio of 0.087% (Table S4). In contrast, the Microbiome kit led to the highest number of mapped SSU reads, averaging 291,246 read pairs per sample with a mapping ratio of 0.14% (Table S4). Interestingly, despite having the lowest SSU mapping ratio, DNeasy F exhibited a higher fraction of completely assembled SSU rRNA sequences, reaching up to 5.76% compared to DNeasy NF, which showed nearly no SSU assembly (Table S4). The Microbiome kit, while achieving the highest SSU mapping ratio, also displayed a substantially larger assembled fraction, averaging 37.1%, suggesting that this method is more efficient to obtain better SSU assemblies (Table S4). These differences in SSU rRNA mapping and assembly efficiency further support the observation that the Microbiome kit enhances more efficient recovery of SSU rRNA sequences compared to the two other methods.

**Fig. 2:**
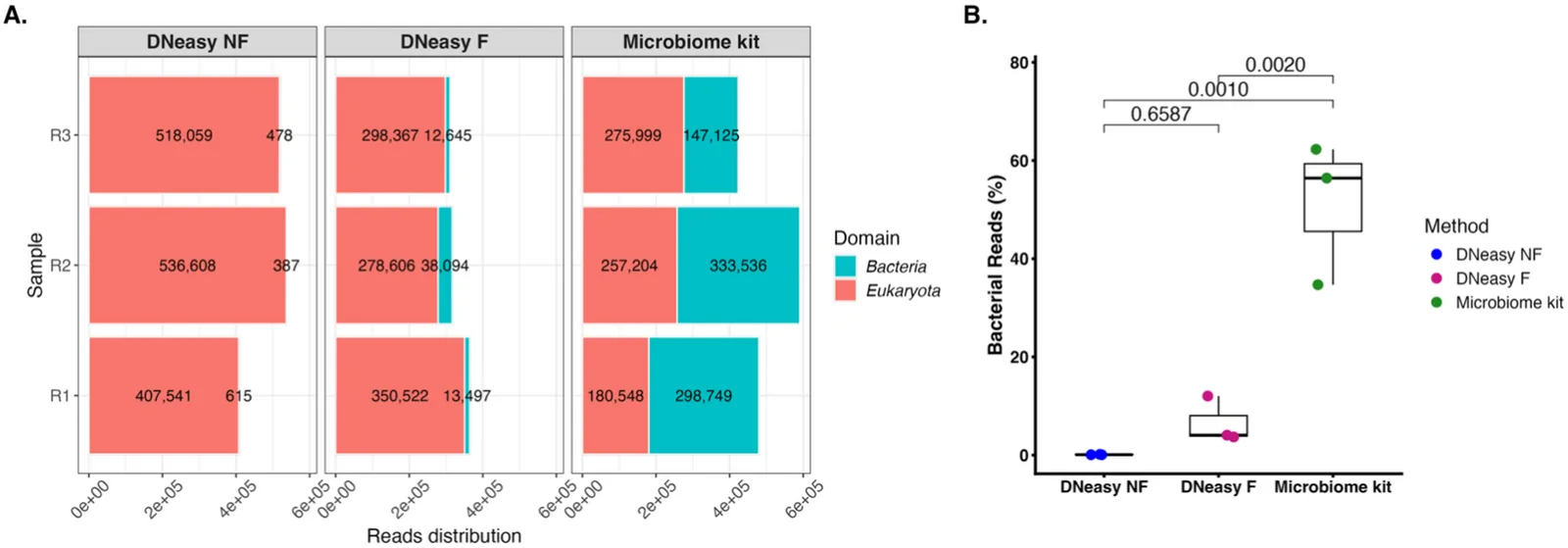
Distribution of bacterial and eukaryotic reads based on SSU rRNA sequence mapping using phyloFlash across different DNA extraction methods. (**A**) Total number of reads successfully mapped to SSU rRNA sequences and assigned to *Eukaryota* (red) and *Bacteria* (blue) for each DNA extraction method (DNeasy NF, DNeasy F, and Microbiome kit) across three replicates (R1, R2, R3). (**B**) Percentage of bacterial SSU rRNA reads per sample. Individual points represent replicates, colour-coded by extraction method (blue for DNeasy NF, pink for DNeasy F, and green for Microbiome kit). Statistical comparisons were performed using ANOVA followed by Tukey’s post-hoc test, with p values above the comparisons

Consistent with the qPCR results, our phyloFlash analysis showed the highest proportion of eukaryotic reads in DNeasy NF and DNeasy F samples (99.9% and 93.4% on average, respectively) compared to the Microbiome kit samples (48.8% on average), with bacterial reads comprising the remaining fraction (Fig. 2, Figure S1A). A one-way ANOVA confirmed a significant effect of DNA extraction method on the percentage of bacterial reads (F = 29.8, p value = 0.0008; Table S5 – Sheet 1). Tukey’s post-hoc test further revealed that the proportion of bacterial reads obtained with the Microbiome kit was significantly higher than that obtained with DNeasy NF (p value = 0.0010) and DNeasy F (p value = 0.0020), whereas no significant difference was observed between the DNeasy NF and DNeasy F methods (p value = 0.6587) (Fig. 2B, Table S5 – Sheet 1).

PhyloFlash taxonomic classification confirmed a high proportion of eukaryotic reads mapped to SSU rRNA sequences in our samples, and the overwhelming majority of these reads was assigned to *Metazoa*. However, the specific limitations of the tool (lack of accounting for the numerous levels of eukaryotic taxonomy, shallow assignation of untargeted SSU sequences) did not allow assigning these sequences to the host *Aedes aegypti* unless the full SSU rRNA gene was assembled (Table S2). To specifically quantify the host-derived fraction of eukaryotic contamination, we mapped these reads onto the *Aedes aegypti* reference genome (AaegL5.0, Figure S1B). This approach allowed us to differentiate mosquito host contamination from other non-host eukaryotic sequences and more accurately assess the efficiency of different DNA extraction methods in reducing host DNA carryover. Consistently, we found that, on average, more than 97% of the eukaryotic reads identified by phyloFlash successfully mapped to the *Aedes aegypti* reference genome, confirming that the vast majority of eukaryotic contamination originates from the host itself (Figure S1B, Table S6).

The Microbiome kit samples yielded the highest number of bacterial NTUs and bacterial reads compared to the other extraction methods (Figure S2). These samples were largely dominated by reads assigned to *Gammaproteobacteria* (~ 89% of bacterial reads on average, well above the levels recovered by either DNeasy method; Figure S2A, Table S2). Importantly, read counts by Phylum confirmed that this pattern is consistent with an additive recovery of *Gammaproteobacteria* rather than a reduction in other bacterial groups, although we note that differential lysis or recovery efficiencies between methods could also contribute to relative abundance shifts, and cross-method relative comparisons should therefore be interpreted with caution (Figure S3). This did not translate into a loss of diversity: Microbiome kit samples recovered > 85% of NTUs detected with the other kits (Figure S4). The Microbiome kit samples also showed a significantly greater number of detected NTUs at the Order level (F = 68.9, Tukey post-hoc, p value = 0.0001 vs. DNeasy NF, p value = 0.0050 vs. DNeasy F; median of ~ 529 vs. ~ 355 vs. ~ 125 NTUs for Microbiome kit, DNeasy F and DNeasy NF respectively; Table S2, Table S5, Figure S2B). Notably, each method also detected its own set of specific NTUs (257 NTUs were uniquely recovered by the Microbiome kit, 72 by DNeasy F and 4 by DNeasy NF), suggesting that each extraction protocol provides access to a partially distinct fraction of the bacterial community (Figure S4A). These patterns suggest that the Microbiome kit enhances the recovery of certain bacterial groups (particularly *Gammaproteobacteria*) while maintaining broad community coverage. Beyond taxonomic composition, the number of bacterial reads per sample also varied significantly across extraction methods (ANOVA + Tukey, p value = 0.0037 Microbiome vs. DNeasy NF; p value = 0.0056 Microbiome vs. DNeasy F; Figure S2C), with a median of ~ 22-fold more bacterial reads in Microbiome kit than in DNeasy F samples (median of ~ 298,700 vs. ~ 13,500 reads) (Figure S1A, Figure S2C). We however note that these observations are based on pooled samples (*n* = 3 per method) and should be read as descriptive of how each extraction method reshapes the recovered community rather than as ecological inferences about midgut microbiota composition.

In contrast, the DNeasy samples showed a more balanced recovered bacterial profile with a dominance of both *Gammaproteobacteria* and *Firmicutes*. The DNeasy F samples showed a slightly distinct profile from the unfiltered DNeasy NF, characterized by a higher relative abundance of *Actinobacteriota* (18.7% of bacterial reads versus 3.7% in DNeasy NF) and, to a lesser extent, of *Gammaproteobacteria* (38.5% versus 24.7% in DNeasy NF, Figure S2A, Table S2, Table S7). Both differential abundance methods (DESeq2 and ALDEx2; Table S7) returned concordant effect sizes identifying *Actinobacteriota* as enriched in DNeasy F. Consistent with the exploratory nature of the pooled design (three replicates per method), we interpret these compositional differences as descriptive rather than as formally significant shifts. The filtration step in DNeasy F likely contributed to this profile by selectively removing bacteria attached to eukaryotic material, adherent to the midgut epithelium, or intracellular, which may explain the lower recovery of certain bacterial groups compared to the other methods. These findings suggest that DNA extraction methods influence not only the proportion of bacterial reads recovered but also the recovered composition and structure of the bacterial community (Figure S2).

These method-dependent differences were further illustrated by NMDS ordination based on Bray-Curtis dissimilarities (stress = 7.518555e-05, Figure S2D), which showed a clear separation between the bacterial NTU profiles obtained with the different extraction methods. PERMANOVA analysis supported this observation, although we interpret it as exploratory given the limited number of pooled replicates (*n* = 3 per method): the DNA extraction method had a significant effect on overall bacterial community structure (R^2^ = 0.89, F = 24.8, p value = 0.001; Table S5, Sheet 2). Group dispersions did not differ significantly across methods (betadisper, F = 0.73, p value = 0.536), indicating that the observed separation is not driven by heterogeneous within-group variance. However, the number of reads per sample did not significantly contribute to this variation (R^2^ = 0.13, F = 1.02, p value = 0.353; Table S5, Sheet 2), suggesting that the observed differences reflect methodological rather than sequencing depth effects.

### Bacterial enrichment and MAG reconstruction estimates using whole genome sequencing reads

To assess the impact of DNA extraction methods on bacterial enrichment and Metagenome-Assembled Genomes (MAGs) reconstruction, we first removed host-derived *Aedes aegypti* reads prior to assembly using the reference genome AaegL5.0. This host DNA depletion step reduced the total number of reads to an average of 67,996,074 paired-end reads (Table S3). Consistent with the qPCR and SSU rRNA taxonomic profiling results, both the number and relative percentage of non-mosquito reads (Figure S5A and Figure S5B, respectively, Table S3) were highest in the Microbiome kit samples (117,025,682 reads on average; 55.35% of total filtered and trimmed reads), followed by the DNeasy F (61,979,290 reads on average; 27.45% of total filtered and trimmed reads), and the DNeasy NF (24,983,249 reads on average; 10.24% of total filtered and trimmed reads) samples. A one-way ANOVA confirmed a significant effect of DNA extraction method on the number of non-mosquito reads, with significantly more of those in Microbiome kit than in DNeasy NF (p value = 0.0032) and DNeasy F (p value = 0.0347) samples, while no significant difference was observed between the two DNeasy methods (p value = 0.1379) (Figure S5A, Table S3). Similarly, the percentage of non-mosquito reads differed significantly across methods (Tukey post-hoc: Microbiome kit vs. DNeasy NF, p value = 0.0010; Microbiome kit vs. DNeasy F, p value = 0.0111; DNeasy NF vs. DNeasy F, p value = 0.0792) (Figure S5B, Table S3).

**Fig. 3:**
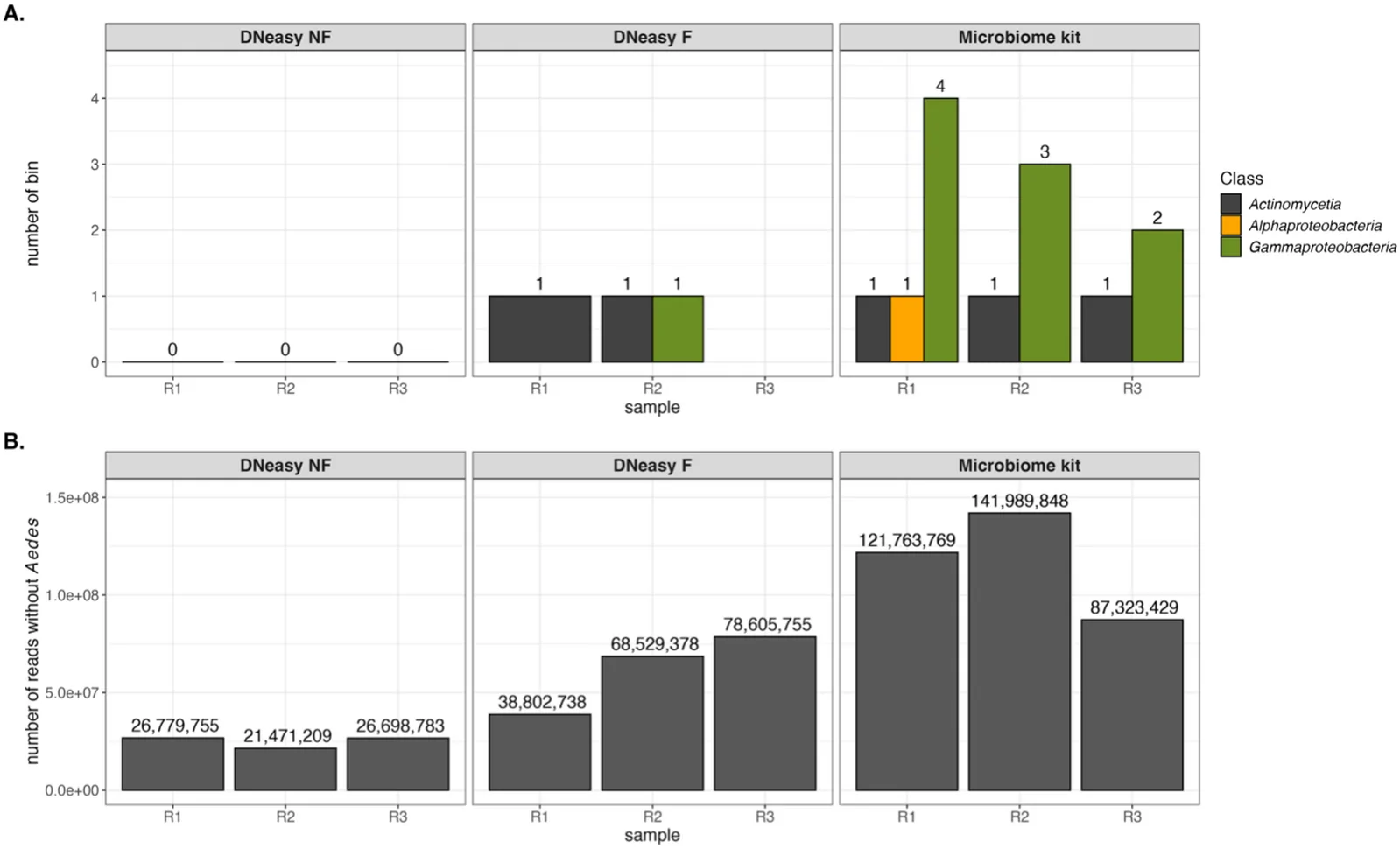
Number of MAGs reconstructed in samples and “non-mosquito” reads for each extraction method. (**A**) Number of high-quality reconstructed MAGs (completion > 90% and redundancy < 10%) at the Class level for each replicate under each DNA extraction condition. (**B**) Number of reads remaining after removal of *Aedes aegypti* reference genome (genome assembly AaegL5.0) for each replicate and DNA extraction condition

We then assessed whether bacterial enrichment strategies could improve MAG reconstruction despite high eukaryotic contamination. After removal of *Aedes aegypti* genome reads to discard host reads, we obtained successful metagenomic assemblies for most samples including those from Microbiome kit and DNeasy F. Only one DNeasy F replicate failed to assemble, likely due to a limited number of bacterial reads, and no MAGs could be reconstructed from DNeasy NF samples (Fig. 3, Table S8). This pattern is consistent with the bacterial enrichment observed across all three analytical approaches (qPCR, SSU rRNA taxonomic profiling and whole-genome sequencing). In DNeasy F samples, we recovered three high-quality MAGs (completion > 90%, redundancy < 10%) including two assigned to *Cutibacterium acnes* and one belonging to the *Enterobacteriaceae* family. The Microbiome kit samples yielded a larger set of 13 MAGs spanning multiple bacterial taxa including the classes *Actinomycetia* (three *Cutibacterium acnes* MAGs), *Alphaproteobacteria* (one assigned to genus *Sphingomonas*) and *Gammaproteobacteria* (Fig. 3, Table S8). The latter included three MAGs belonging to the *Enterobacteriaceae* family, three MAGs to the genus *Achromobacter*, two MAGs to *Pseudomonas*, and one MAG to *Comamonas*, respectively (Fig. 3, Table S8). These results are consistent with the effectiveness of the Microbiome kit in enriching bacterial DNA and facilitating the recovery of high-quality MAGs from mosquito midgut samples.

## Discussion

Overall, we observed significant differences in bacterial enrichment between the filtration and lysis-based DNA extraction methods applied to *Aedes* midguts. Both the DNeasy F and the Microbiome kit samples showed significant bacterial enrichment due to the effective depletion of host mosquito DNA (*RpS30*, Fig. 1B) either by tissue filtration (DNeasy F) or by differential cell lysis (Microbiome kit). Among the methods tested, the Microbiome kit was the most effective for enriching bacterial DNA and facilitating bacterial MAG reconstruction, although it showed a compositional preference toward *Gammaproteobacteria*. Importantly, this occurred without a loss of overall bacterial diversity, as it still captured > 85% of the NTUs detected with the other kits. The filtration step prior to extraction with the DNeasy kit also reduced eukaryotic contamination and enabled the recovery of some MAGs, though less efficiently than the Microbiome kit. It however yielded a slightly divergent community profile and recovered 72 method-specific NTUs (alongside 257 for the Microbiome kit and 4 for DNeasy NF; Figure S4A), illustrating that the methods are not strictly nested in their taxonomic coverage and that no single method captures the full bacterial NTU diversity. Together, these findings suggest that combining complementary extraction strategies may improve microbiota analyses from host-associated, and especially low-biomass samples such as mosquito midguts.

Among the reconstructed MAGs, *Cutibacterium acnes* was identified in two DNeasy F samples and in all three Microbiome kit samples. This bacterium is closely associated with human samples and may originate from human skin contamination during laboratory handling. Other bacterial MAGs reconstructed from the Microbiome kit samples included taxa such as *Enterobacteriaceae*, *Pseudomonas*, *Sphingomonas*, *Achromobacter* and *Comamonas* which have been previously reported as potential contaminants from kitomes [4, 25, 31, 33, 39, 67, 68]. Without strain-level evidence, and without environmental contamination controls beyond our blanks, these taxa should be read as ambiguous (plausible kit- or lab-derived contaminants) but also possibly genuine components of mosquito-associated microbiota, as reported in the mosquito microbiome literature [69–72]. Our blanks did not show comparable 16S signal, which argues against systematic kit contamination, but does not exclude it entirely.

Interestingly, the comparison between the DNeasy kit samples with and without filtration confirmed that filtration significantly depletes host mosquito DNA (*RpS30*, Fig. 1B). However, the low number of MAGs reconstructed from DNeasy F samples suggests that while filtration helps depleting host DNA, it may also lead to a reduction in bacterial yield, limiting MAG reconstruction. This reduction is consistent with our qPCR results, which showed lower bacterial abundance in DNeasy F samples compared to DNeasy NF and Microbiome kit. A likely explanation is that some bacteria were lost during the filtration step, possibly because they remained attached to eukaryotic material, adhered to the midgut epithelium, or were intracellular. At the same time, lysis efficiency with the DNeasy kit may have been lower, meaning that some taxa, including Gram-negative *Gammaproteobacteria*, which possess an outer membrane, were better recovered by the Microbiome kit. The higher proportion of *Gammaproteobacteria* observed with the Microbiome kit is actually consistent with additive recovery of these taxa rather than a compositional bias, as read counts by Phylum confirmed that other bacterial groups were not reduced in absolute terms (Figure S3). That said, differential lysis or recovery efficiencies between methods could also contribute to relative abundance shifts [73], and cross-method relative comparisons should be interpreted with caution. Considering that DNeasy NF also recovered *Gammaproteobacteria*, although at lower relative levels than the Microbiome kit, it is likely that both processes, filtration loss and differential lysis efficiency, contributed to the observed differences. In contrast, the Microbiome kit did not require filtration yet effectively reduced host contamination while maintaining a higher yield of bacterial reads. The higher proportion of *Gammaproteobacteria* observed with this method could reflect either selective recovery of these taxa or, alternatively, reduced recovery of them with the DNeasy protocols.

Further analyses with a larger sample size could potentially determine whether the Microbiome kit systematically alters the microbiome composition recovery. Overall, combining different DNA extraction methods, with or without filtration, may provide a more comprehensive view and approach for reconstruction of bacterial genomes associated with mosquito midguts.

## Conclusion

Our study shows the significant impact of DNA extraction methods on bacterial enrichment, profiling, and MAG reconstruction in midgut tissues. The Microbiome kit was the most effective at minimizing host DNA and maximizing bacterial recovery, enabling the reconstruction of a larger and more taxonomically varied set of MAGs, although with a compositional shift towards *Gammaproteobacteria*. This occurred without loss of overall bacterial diversity and is consistent with improved recovery of these taxa compared to other extraction methods rather than a compositional bias. The DNeasy kit with manual filtration reduced eukaryotic contamination and yielded a more balanced recovered community profile but enabled the reconstruction of fewer MAGs than the Microbiome kit. Notably, each method also recovered a fraction of method-specific NTUs, reinforcing the rationale for combining extraction strategies. Overall, host DNA depletion proved to be an effective way to increase the bacterial sequence recovery, with the Microbiome kit performing best among the tested methods. These findings underscore the importance of selecting an appropriate extraction method based on the research objective, whether prioritizing bacterial community profiling, host DNA depletion, or genome reconstruction. A combined approach integrating different extraction techniques may provide a more comprehensive and reproducible characterization of midgut microbiomes, ultimately improving metagenomic studies of host-associated microbial communities and other low-biomass samples. Finally, these host-depletion strategies, particularly the Microbiome kit, markedly increased bacterial recovery from pooled midguts. Whether these gains are sufficient to enable microbiome analyses at the single-midgut level, which would reduce the need for pooling and restore access to inter-individual variation, remains to be tested and represents a promising direction for future work.

## Supplementary Information

Below is the link to the electronic supplementary material.

Supplementary Material 1

Supplementary Material 2

Supplementary Material 3

Supplementary Material 4

Supplementary Material 5

Supplementary Material 6

Supplementary Material 7

Supplementary Material 8

Supplementary Material 9

Supplementary Material 10

Supplementary Material 11

Supplementary Material 12

Supplementary Material 13

Supplementary Material 14

## Data Availability

The raw sequences can be accessed in the European Nucleotide Archive database (Study accession number: PRJEB96753). The merged anvi’o profiles databases are available in Zenodo (https://doi.org/10.5281/zenodo.16971557). A reproducible bioinformatics workflow including scripts used to perform all the computational analyses (statistics, figures, metagenomics workflow) is available here: https://github.com/HansSCHR/bacterial_enrichment_mosquito. These resources allow users to fully reproduce all computational analyses presented in this study.
