# Supplementary material for: Optimizing bacterial enrichment procedures to improve mosquito midgut microbiome research
Source: Anim Microbiome. 2026 Aug 17;8:120. doi: 10.1186/s42523-026-00614-0 (PMC13757207; doi:10.1186/s42523-026-00614-0)
Supplement: Supplementary file 1 [file 42523_2026_614_MOESM6_ESM.docx]

Supplementary Information

**Table S1. Real-time quantitative PCR (qPCR) data**. **Sheet 1**. Individual data for the 114 assigned wells: well position, sample, extraction condition, replicate, target (*RpS30* or 16S rRNA), task, cycle threshold (CT), amplification status and melting temperatures. Wells that did not amplify within the 40 cycles of the run are reported as "Undetermined"; twelve wells excluded as technical outliers during the original analysis are left empty. **Sheet 2**. Mean CT, standard deviation and number of wells used, per sample and target, calculated from the amplifying wells in Sheet 1.

**Table S2. Taxonomic profiling based on SSU rRNA sequences (phyloFlash)**. Taxonomic summaries based on the mapping hits of the quality filtered reads to the SILVA SSU rRNA database performed during the phyloFlash analyses for each sample from each extraction condition (Sheet 1-9). The table reports all the identified taxa (from domain to species rank) and corresponding number of reads.

**Table S3. Quality filtering and assembly statistics** for each sample from each extraction condition. Sequencing name, sample name, total number of raw pairs of reads, number of filtered pairs of reads, percent of filtered (passed) reads, number of filtered pairs of reads after trimming, total pairs reads after removing the *Aedes aegypti* genome, percent of *Aedes aegypti* and non-*Aedes aegypti* genome reads, number of contigs > 1kb, L50, N50 and percent of high-quality recruited reads by sample.

**Table S4. Report of phyloFlash mapping and reconstruction of SSU rRNA sequences.** Sequencing name, sample name, number of input paired-end reads for phyloFlash, number of mapped SSU read pairs, percent of mapped SSU read pairs, percent of assembled reads into full-length SSU rRNA sequence, detected median insert size, insert size standard deviation.

**Table S5. Statistical analyses of the effect of DNA extraction methods on eukaryotic contamination, bacterial read abundance, diversity, and community composition.** This table reports the results of statistical tests evaluating the impact of DNA extraction methods on different aspects of microbiome composition in mosquito midgut samples. **Sheet 1.** Effect of DNA extraction method on the percentage of eukaryotic and bacterial reads, number of bacterial reads, number of detected bacterial NTUs at the Order level (ANOVA and Tukey’s post-hoc test results). **Sheet 2.** NMDS ordination based on Bray-Curtis dissimilarity distance, PERMANOVA results testing the influence of extraction method, total read count and their combined effect on bacterial community composition, and a permutational test for homogeneity of multivariate dispersions (betadisper).

**Table S6. Percent of eukaryotic reads identified using phyloFlash mapped or unmapped onto *Aedes aegypti* reference genome (genome assembly AaegL5.0, NCBI ID: GCF_002204515.2).** Sequencing name, Method (sample), Replicate, Category (mapped or unmapped label), percent of mapped or unmapped eukaryotic reads.

**Table S7. Differential abundance of bacterial taxa between DNA extraction methods.** Bacterial read counts (phyloFlash; Table S2) were compared between each pair of extraction methods with two complementary approaches, as described in the Methods: DESeq2 (negative binomial model) and the compositional method ALDEx2 (centered log-ratio transformation), each with Benjamini-Hochberg correction. The table reports the log2 fold change and adjusted p-value (padj) for DESeq2, and the standardized effect size and adjusted p-value for ALDEx2. For both methods, positive values indicate a higher abundance in the first extraction method of the comparison. NA indicates that DESeq2 did not return an adjusted p-value for that taxon (very low counts). Given the pooled design and three replicates per method, these analyses remain exploratory.

**Table S8. Refined bacterial MAGs estimates** for each sample from each extraction condition. The table reports for each bin: sample id, extraction condition (DNeasy NF, DNeasy F and Microbiome kit), bins, total number of nucleotides, number of contigs, N50, GC content, completion and redundancy rates, and taxonomy (from domain to species level). **Sheet 1** reports every bin obtained with CONCOCT (n = 747) and **Sheet 2** reports the 16 high-quality MAGs retained for the analysis (completion > 90%, redundancy < 10%).

**Figure S1.** **Distribution of bacterial and eukaryotic reads based on SSU rRNA sequence mapping using phyloFlash across different DNA extraction methods.** (A) Relative abundance of mapped SSU rRNA reads assigned to *Eukaryota* (red) and *Bacteria* (blue) using phyloFlash for each DNA extraction method and replicates. (B) Pie charts showing the proportion of eukaryotic SSU rRNA reads that successfully mapped to the *Aedes aegypti* reference genome (AaegL5.0; dark red) and those that remained unmapped (grey) for each method and replicate. Percentages indicate the mapped and unmapped fractions.

**Figure S2. Bacterial NTU composition and diversity across DNA extraction methods based on SSU rRNA sequences using phyloFlash.** (A) Relative abundance of reads assigned to bacterial NTUs from SSU rRNA sequences using phyloFlash at the *Phylum* level across the three DNA extraction methods (DNeasy NF, DNeasy F, and Microbiome kit). Each bar represents one replicate (R1, R2, R3), and colours correspond to different bacterial phyla. (B) Number of detected bacterial NTUs at the *Order* level for each DNA extraction method. Violin and box plots show the distribution of detected NTUs per sample, with statistical comparisons performed using ANOVA followed by Tukey’s post-hoc test. Significant differences are indicated by p values above the comparisons. (C) Total number of bacterial reads assigned to bacterial SSU rRNA sequences per replicate by method. The y-axis is log-transformed (base 10) to enhance clarity. Statistical comparisons were performed using ANOVA followed by Tukey’s post-hoc test, and significant differences are reported above the comparisons. (D) NMDS ordination (Bray-Curtis) of bacterial community composition across DNA extraction methods. Each point represents a replicate, coloured by extraction method (blue: DNeasy NF, pink: DNeasy F, green: Microbiome kit).

**Figure S3.** **Bacterial NTU count across DNA extraction methods based on SSU rRNA sequences using phyloFlash.** Reads count of bacterial NTUs identified from SSU rRNA sequences using phyloFlash at the *Phylum* level across the three DNA extraction methods (DNeasy NF, DNeasy F, and Microbiome kit). Each bar represents one replicate (R1, R2, R3), and colours correspond to different bacterial phyla.

**Figure S4. Comparison of NTU overlap and read distribution across extraction methods.** (A) UpSet plot showing the number of NTUs shared or unique across the three extraction methods (DNeasy NF, DNeasy F, and Microbiome kit). Barplots indicate the size of each NTUs intersection (top) and total NTUs set size (left). (B) Relative abundance of reads assigned to NTUs (%) falling into each intersection category. Most reads were assigned to NTUs shared by all three methods. (C) Absolute number of reads assigned to NTUs for each method, with separate y-axes to account for differences in sequencing depth. To note, the "Other" category includes NTUs present only in DNeasy NF or shared between subsets of methods not explicitly represented in the main categories.

**Figure S5. Non-mosquito DNA material enrichment between different DNA extraction methods.** (A) Number of reads remaining after removal of *Aedes aegypti* reference genome reads (genome assembly AaegL5.0) per DNA extraction methods. (B) Relative abundance of non-mosquito reads after removal of *Aedes aegypti* genome reads per DNA extraction methods. N = 3 samples of 5 midguts per group. The black bars display the medians. Data were analyzed with one-way ANOVA followed by a Tukey’s multiple comparison test. Exact p values are shown.
